# Assessing the Role of Pharyngeal Cell Surface Glycans in Group A *Streptococcus* Biofilm Formation

**DOI:** 10.3390/antibiotics9110775

**Published:** 2020-11-04

**Authors:** Heema K. N. Vyas, Anuk D. Indraratna, Arun Everest-Dass, Nicolle H. Packer, David M. P. De Oliveira, Marie Ranson, Jason D. McArthur, Martina L. Sanderson-Smith

**Affiliations:** 1 Illawarra Health and Medical Research Institute, Wollongong 2522, Australia; hv997@uowmail.edu.au (H.K.N.V.); anuk@uow.edu.au (A.D.I.); mranson@uow.edu.au (M.R.); 2 School of Chemistry and Molecular Bioscience, Molecular Horizons, University of Wollongong, Wollongong 2522, Australia; jasonm@uow.edu.au; 3 Institute for Glycomics, Griffith University, Southport 4215, Australia; a.everest-dass@griffith.edu.au (A.E.-D.); nicki.packer@mq.edu.au (N.H.P.); 4 Department of Molecular Sciences, Macquarie University, Sydney 2109, Australia; 5 School of Chemistry and Molecular Biosciences, University of Queensland, Brisbane 4072, Australia; d.deoliveira@uq.edu.au

**Keywords:** group A *Streptococcus*, *Streptococcus pyogenes*, biofilm, glycans, antibiotics, penicillin, EPS

## Abstract

Group A *Streptococcus* (GAS) causes 700 million infections and accounts for half a million deaths per year. Antibiotic treatment failure rates of 20–40% have been observed. The role host cell glycans play in GAS biofilm formation in the context of GAS pharyngitis and subsequent antibiotic treatment failure has not been previously investigated. GAS serotype M12 GAS biofilms were assessed for biofilm formation on Detroit 562 pharyngeal cell monolayers following enzymatic removal of all *N*-linked glycans from pharyngeal cells with PNGase F. Removal of *N*-linked glycans resulted in an increase in biofilm biomass compared to untreated controls. Further investigation into the removal of terminal mannose and sialic acid residues with α1-6 mannosidase and the broad specificity sialidase (Sialidase A) also found that biofilm biomass increased significantly when compared to untreated controls. Increases in biofilm biomass were associated with increased production of extracellular polymeric substances (EPS). Furthermore, it was found that M12 GAS biofilms grown on untreated pharyngeal monolayers exhibited a 2500-fold increase in penicillin tolerance compared to planktonic GAS. Pre-treatment of monolayers with exoglycosidases resulted in a further doubling of penicillin tolerance in resultant biofilms. Lastly, an additional eight GAS *emm*-types were assessed for biofilm formation in response to terminal mannose and sialic acid residue removal. As seen for M12, biofilm biomass on monolayers increased following removal of terminal mannose and sialic acid residues. Collectively, these data demonstrate that pharyngeal cell surface glycan structures directly impact GAS biofilm formation in a strain and glycan specific fashion.

## 1. Introduction

Group A *Streptococcus* (*Streptococcus pyogenes*; GAS) is a Gram-positive human pathogen responsible for a variety of infections. GAS pharyngitis is the most common disease state, with global incidence estimated at 600 million cases per year [1]. Currently, penicillin remains the antibiotic of choice for treating GAS infections, with no reports of penicillin resistance among clinical GAS isolates. However, antibiotic treatment failure has been well-documented at rates of 20–40% for GAS pharyngitis [2,3,4]. Biofilms enable bacteria to survive and tolerate both host immunity and antimicrobial treatment. As a result, biofilm-associated infections are oftentimes chronic and recurrent, and particularly difficult to clear and treat [3,4,5]. Recently, it has been proposed that GAS may form biofilms, contributing to antibiotic treatment failure [3]. There is evidence of bacterial biofilm formation in human tonsils [6], and in a study of patients suffering from recurrent GAS pharyngo-tonsillitis, the presence of GAS biofilms in tonsillar crypts was identified in all 30 patients [7]. Furthermore, in a study assessing GAS isolated from pharyngitis patients non-responsive to antibiotics, all 99 GAS isolates demonstrated biofilm-forming ability in vivo, with 60% of these displaying increased penicillin tolerance once in the biofilm phenotype [3]. However, most of these studies utilized abiotic surfaces (glass, plastic, polystyrene) and very few incorporated surface coating with extracellular matrix components such as fibronectin, fibrinogen, laminin, or collagen [8,9,10,11]. The host cell surface is integral in initial host-pathogen interactions, bacterial cell adherence, and subsequent biofilm formation [12]. Thus, there is a need to better understand the role of host cell surface receptors in GAS biofilm formation.

Glycans are carbohydrates present on more than half of all human proteins, with their ubiquitous presence in mucosal fluid, on secreted molecules, immune cells, and a variety of epithelial cell surfaces, making glycosylation one of the most common post-translational modifications [13,14]. Glycans are the initial point of contact with the host for many bacteria, mediating attachment and colonization, and can consequently facilitate infection and disease. Several human bacterial pathogens such as *Pseudomonas aeruginosa*, *Enterococcus faecalis*, *Helicobacter pylori*, *Streptococcus gordonii*, and *Streptococcus pneumoniae* have proven adept at adhering to and utilizing glycan structures for pathogenesis [15,16,17,18,19]. Bacterial modification of host glycan structures enables binding to otherwise inaccessible host receptors for adherence, modulation of host immune molecules, acquisition of carbohydrate substrates for non-glucose fermentation, facilitation of interspecies competition, and promotion of biofilm formation [15,16,17,18,19]. An example of a well-characterized glycan-utilizing system is the NanA neuraminidase of *S. pneumoniae,* which cleaves off terminal sialic acid residues from glycans. NanA has been shown to be particularly active during cluster formation and biofilm maturation in vivo [20]. Another study found increased *S. pneumoniae* biofilm formation was seen only for biofilms exposed to sialic acid, but not in the presence of other glycans [21].

The utilization of glycans by GAS remains poorly understood, despite GAS possessing an array of adhesins capable of binding structures on the richly glycosylated epithelial surfaces and in the fluids of the oropharynx. Studies to date have focused on planktonic GAS-host glycan interactions in the context of the M protein, a major surface-expressed protein which has a role in the adherence of GAS to host tissues [22,23,24,25]. Investigation of the ability of M proteins from 49 GAS serotypes to associate with various pharyngeal- and dermal-associated glycosaminoglycans (GAGs) revealed that GAGs mediated GAS adhesion to human cells in an M protein-dependent manner [23]. Recently, M1 GAS was shown to bind glycans via the M protein, including the ABO(H) blood group antigens, which are oligosaccharides abundant on the epithelia of most individuals, including the pharynx [24]. Similar trends were observed in two other prevalent GAS M-types, M3 and M12 [25]. Interestingly, this study showed that modification of the cell surface glycome via treatment with a range of exoglycosidases altered GAS-host cell interactions [25]. Taken together, these findings further highlight the role of glycans in mediating GAS adherence.

Whilst the role of host glycans in GAS adherence has been investigated, their role in GAS biofilm formation has yet to be examined. Investigation of glyco-interactions at the GAS-tissue interface in the context of biofilms will enhance our understanding of GAS biofilms and GAS pathogenesis. Consequently, findings of such studies may inform and support the development of novel anti-biofilm strategies as well as biofilm-specific antibiotic treatments. Herein, we examine the role of human pharyngeal *N*-glycans in M12 GAS biofilm formation and subsequent penicillin tolerance.

## 2. Results

### 2.1. Indiscriminate Removal of N-linked Glycans from the Pharyngeal Cell Surface Via PNGase F Treatment Results in Increased M12 GAS Biofilm Biomass

**Figure 1:**
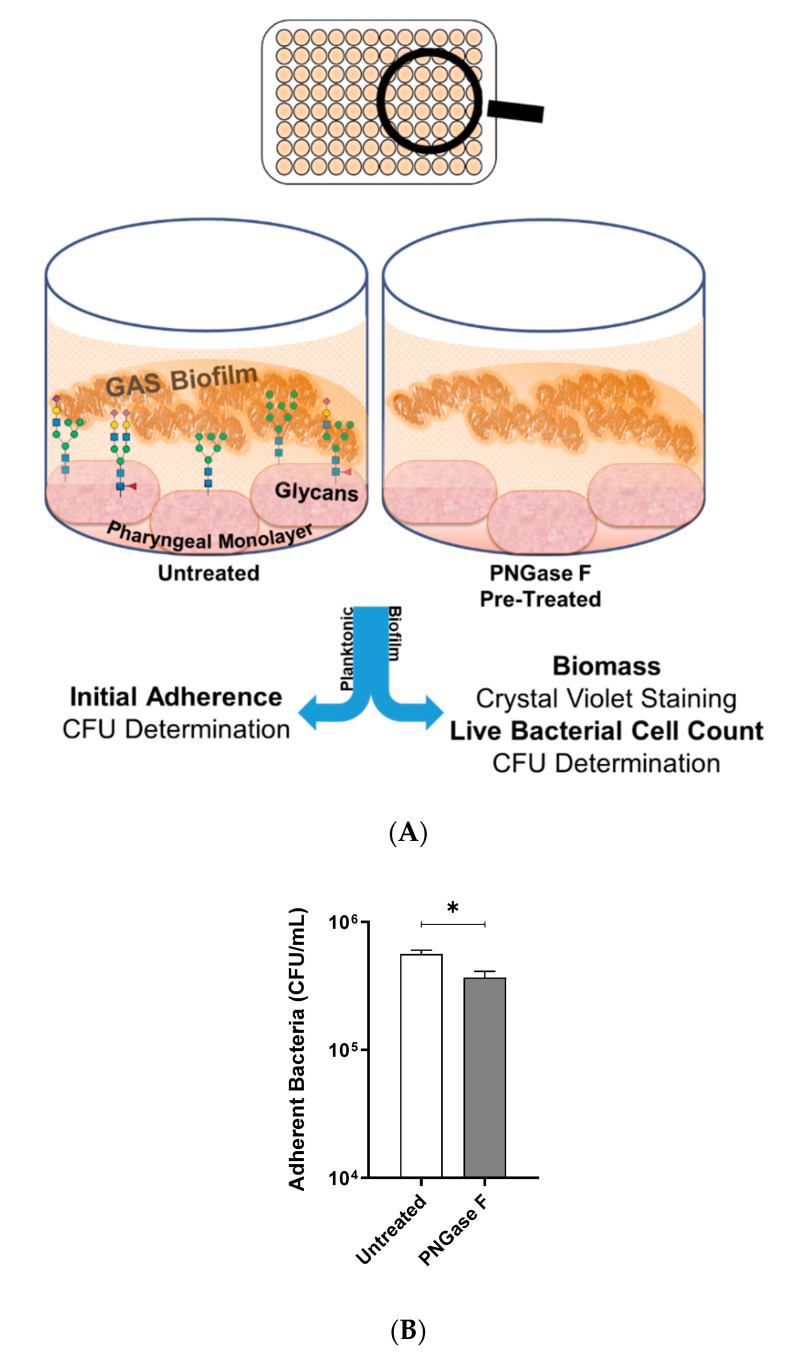

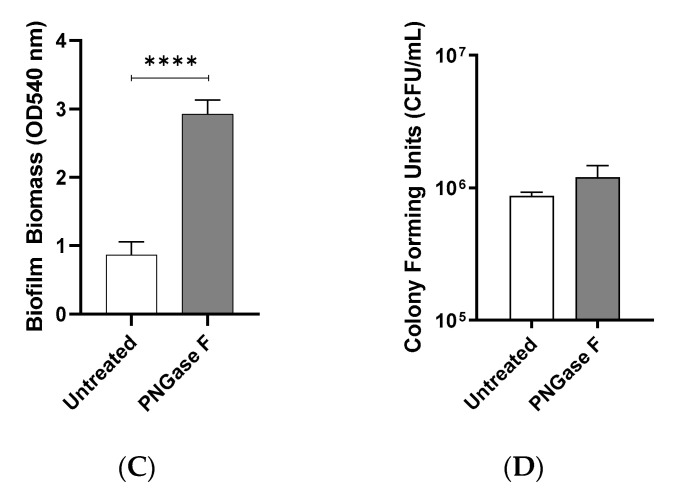
Indiscriminate removal of *N*-linked glycans from the pharyngeal cell surface via PNGase F treatment results in increased M12 Group A *Streptococcus* (GAS) biofilm biomass. (**A**) Assay schematic for 72 h M12 GAS biofilms formed on PNGase F pre-treated and untreated pharyngeal monolayers. (**B**) Initial adherence enumerated for planktonic GAS following 2 h incubation with Detroit-562 cell monolayers. 72 h biofilms were assessed for (**C**) biofilm biomass via crystal violet staining and (**D**) colony forming units via enumeration. Data represents mean ± SEM, with statistical analysis performed, * (*p* ≤ 0.05) and **** (*p* ≤ 0.0001); *n* = 3 biological replicates, with 3 technical replicates each.

M12 GAS, an M-type frequently associated with GAS pharyngitis [26,27], was investigated for its biofilm-forming ability on Detroit 562 pharyngeal monolayers following removal of *N*-linked glycans via peptide-*N*-glycosidase F (PNGase F) treatment. Initial adherence of planktonic M12 GAS was determined by colony forming units, and biofilms were assessed for biofilm biomass and live bacterial cell count by crystal violet staining and colony forming units determination, respectively (Figure 1A). In the absence of *N*-linked glycans, planktonic M12 GAS displayed a significant decrease in initial adherence (*p* ≤ 0.05) when compared to adherence to untreated cells (Figure 1B). Despite a reduction in initial GAS adherence, M12 GAS biofilm biomass increased significantly (*p* ≤ 0.0001) on the surface of PNGase F treated cells compared to the untreated control (Figure 1C). However, PNGase F pre-treatment of monolayers did not significantly affect the GAS CFU within the biofilms (Figure 1D).

**Figure 2:**
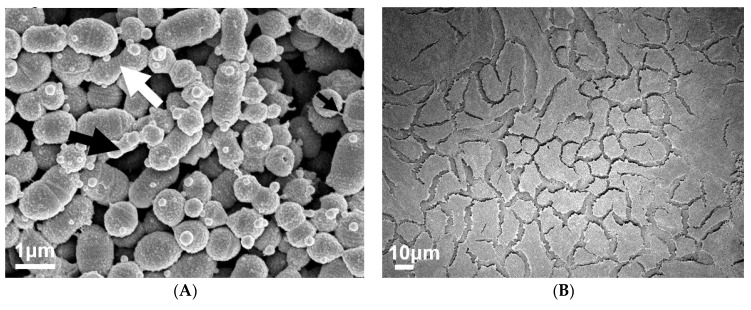

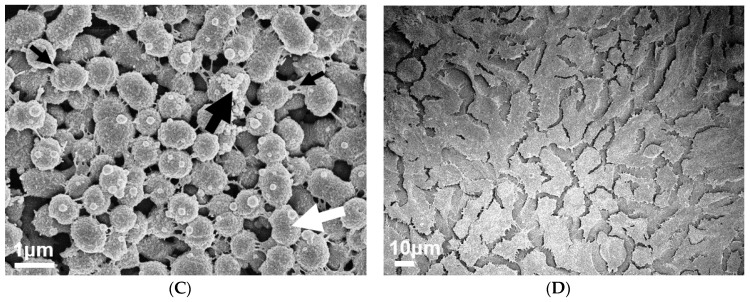
Visual inspection of 72 h M12 GAS biofilms captured via SEM revealed substantial extracellular polymeric substances (EPS) present in biofilms formed on PNGase F pre-treated pharyngeal cell monolayers. Images are representative of biofilms formed on (**A**) untreated and (**C**) PNGase F pre-treated pharyngeal monolayers. GAS biofilms show chained cocci (white arrows) arranged into three dimensional aggregated structures with EPS matrix material present (big and small black arrows). SEM images of (**B**) untreated and (**D**) PNGase F pre-treated Detroit 562 pharyngeal cell monolayers (without biofilm) are also included. Biofilms and Detroit 562 pharyngeal cell monolayers (without biofilm) were imaged using the JEOL JSM-7500 microscope at 15,000× and 500× magnification, respectively. SEM images were randomly selected and represent two biological replicates with two technical replicates each.

To visually investigate the effect of PNGase F pre-treated pharyngeal monolayers on M12 GAS biofilm formation, SEM imaging was conducted. Biofilms formed on both untreated and PNGase F pre-treated monolayers show M12 GAS chained cocci arranged into three dimensional aggregated structures with extracellular polymeric substances (EPS) matrix material present (Figure 2). Biofilms formed on PNGase F pre-treated pharyngeal monolayers (Figure 2C) appear to have more EPS matrix compared to biofilms formed on untreated cells (Figure 2A). EPS matrix material is associated with the aggregated GAS cocci. Interestingly, the EPS produced on the GAS biofilms seemed to come in two distinct forms, a web-like mesh matrix (small black arrows) and a more globular matrix (large black arrows).

Untreated and PNGase F pre-treated Detroit 562 pharyngeal monolayers (without biofilm) were also imaged as controls to ensure that PNGase F treatment did not affect pharyngeal cell morphology (Figure 2B,D).

### 2.2. Characterization of N-linked Glycans from Detroit 562 Pharyngeal Cell Surface Reveals Abundance of Mannose and Sialic Acid Terminating Glycan Structures

To further examine the role of host *N*-glycans on the surface of pharyngeal monolayers in GAS biofilm formation, a comprehensive profile of the Detroit 562 pharyngeal cell surface *N*-glycome was determined. Membrane proteins were purified from pharyngeal cell culture lysates and treated with PNGase F. Released *N*-glycans were identified using porous graphitised carbon liquid chromatography (PGC-LC) and tandem mass spectrometry by electrospray ionisation (ESI-MS/MS). A total of 19 unique structures were detected, not including linkage isomers (see Supplementary Material, Table S1).

**Figure 3:**
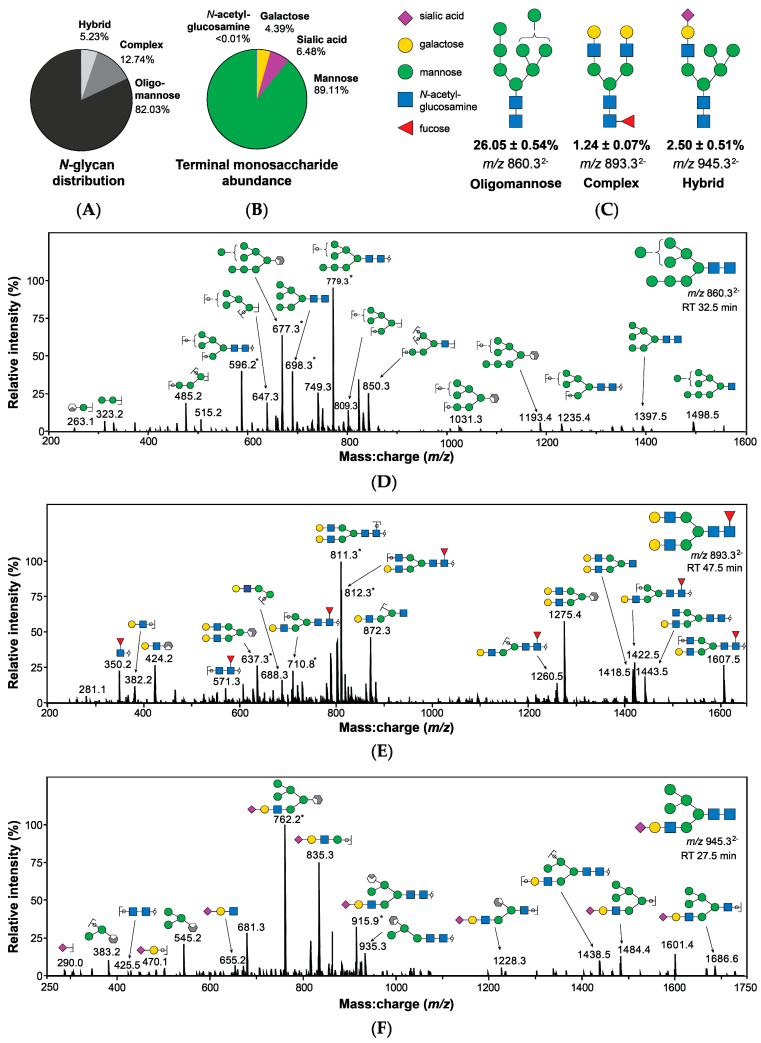
Structures bearing terminal mannose predominate the surface of Detroit 562 pharyngeal cells as determined by PGC-LC-ESI-MS/MS. (**A**) Surface *N*-glycans are primarily oligomannose structures. (**B**) Mannose is the most abundant terminal monosaccharide of *N*-glycans. (**C**) Examples of oligomannose, complex, and hybrid *N*-glycans are provided, including relative abundance and mass-to-charge ratio (*m*/*z*) as detected by MS. Structures were identified primarily using MS^2^ spectra (**D**–**F**) [28,29,30,31] in addition to precursor mass:charge ratio (*m*/*z*) and retention time. Structural isomers sharing the same *m*/*z*, composition, and terminal monosaccharide presentation were combined in evaluation of abundance, calculated by integration of area under of the curve from extracted ion chromatograms. Abundance values are relative and are presented as combined mean ± SEM from 3 biological replicates, each with 3 technical replicates. * Denotes doubly charged fragments. Glycans are represented using conventional graphical nomenclature [32].

Quantitation by relative abundance revealed oligomannose structures to be the predominant class of *N*-glycans, comprising 82.03% of the Detroit 562 pharyngeal cell surface *N*-glycome (Figure 3A) with further analyses confirming that mannose was by far the most abundant terminal monosaccharide, followed by sialic acid and galactose (Figure 3B). Terminal *N*-acetylglucosamine was detected at a very low abundance (<0.01%). Core fucosylation was observed in 14% of structures. Glycans were identified based primarily on MS^2^ fragmentation data with representative spectra of the most abundant *N*-glycans of each class (Figure 3C) provided in Figure 3D–F.

### 2.3. Removal of Terminal Mannose and Sialic Acid Residues from Pharyngeal Cell Surface Glycans Differentially Impacts the Capacity of M12 GAS to Form Biofilm

#### 2.3.1. Initial Adherence, Biofilm Biomass, and Bacterial Colony Forming Units

Oligomannose, sialic acid, and galactose were found to comprise the major *N*-linked glycans on the surface of Detroit 562 pharyngeal cells. Sialic acid is utilised by several pathogens at the nasopharynx in colonisation and biofilm formation [20,21]. Due to the abundance of mannose, and the previously described importance of sialic acid in bacterial virulence, the role of these structures in GAS biofilm formation was further explored.

**Figure 4:**
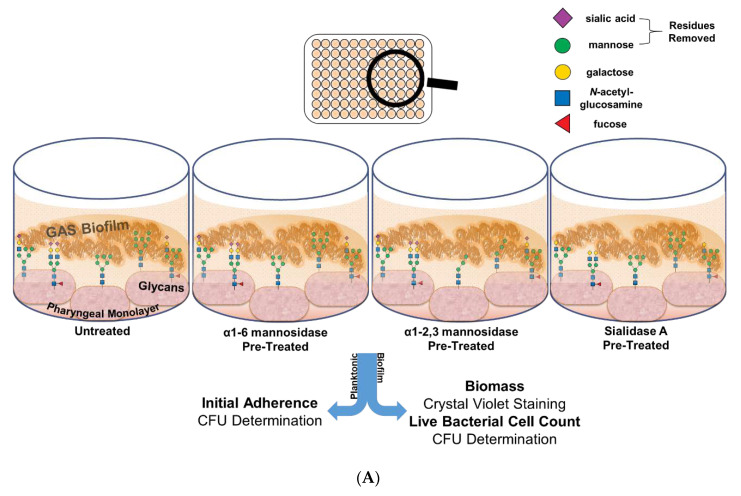

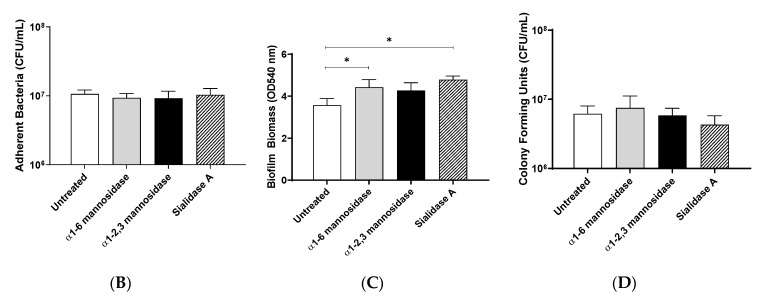
Pre-treatment of pharyngeal cell surface with α1-6 mannosidase and Sialidase A results in significantly increased M12 GAS biofilm biomass. (**A**) Assay schematic for the characterization of biofilms formed on each of the exoglycosidase (α1-6 mannosidase, α1-2,3 mannosidase, and Sialidase (A) pre-treated pharyngeal monolayers vs. untreated. (**B**) Initial adherence enumerated for planktonic GAS upon 2 h incubation. 72 h biofilms are assessed for (**C**) biofilm biomass via crystal violet staining and (**D**) colony forming units via enumeration. Data represents mean ± SEM, with statistical analysis performed using a one-way ANOVA with Tukey’s multiple comparisons test * (*p* ≤ 0.05); *n* = 3 biological replicates, with 3 technical replicates each.

To do so, Detroit 562 pharyngeal monolayers were first pre-treated with specific exoglycosidases; α1-6 mannosidase (removes α1-6 linked mannose residues), α1-2,3 mannosidase (removes α1-2 and α1-3 linked mannose residues), and the broad specificity sialidase (which will be referred to as Sialidase A) (removes linear and branched terminal α2-3, α2-6, α2-8, and α2-9 linked sialic acid); and monosaccharide removal was confirmed by lectin binding assays (see Supplementary Material, Figure S2). Initial adherence of planktonic GAS after 2 h incubation with the untreated and exoglycosidase pre-treated monolayers was determined. The biofilms formed on these monolayers were assessed for biofilm biomass and bacterial colony forming units (Figure 4A).

Investigation into the initial adherence of planktonic M12 GAS interacting with the exoglycosidase pre-treated and control monolayers found no significant differences (Figure 4B). Despite this, the 72 h M12 GAS biofilms exhibited increased biomass on the exoglycosidase pre-treated monolayers compared to the untreated control (Figure 4C). Notably, α1-6 mannosidase and Sialidase A pre-treatment of monolayers resulted in significant increases (*p* ≤ 0.05) in biofilm biomass. The number of colony forming units within the biofilms did not differ significantly between treatments (Figure 4D).

#### 2.3.2. Biofilm EPS

**Figure 5:**
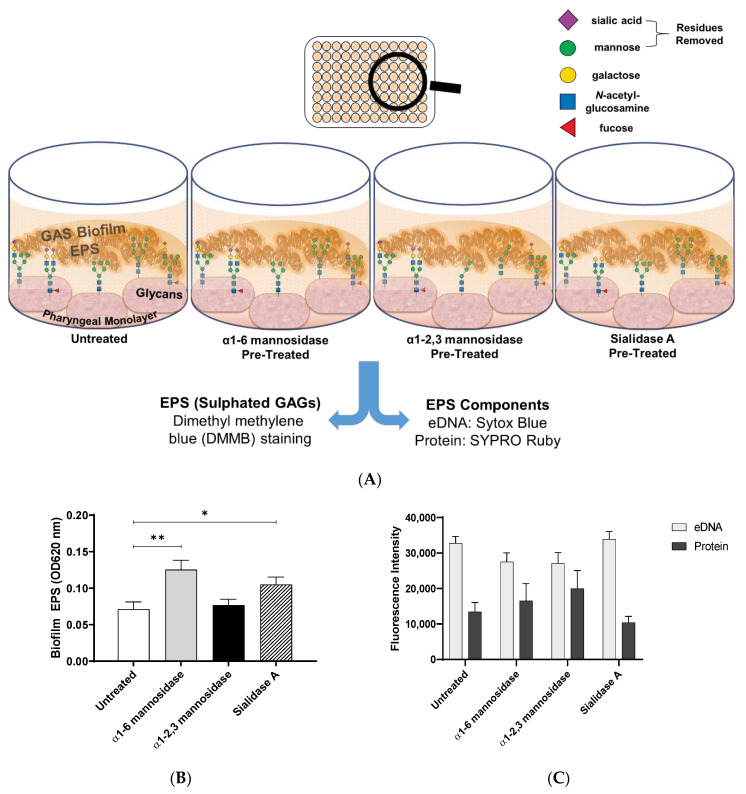
Biofilm EPS increases significantly for biofilms formed on α1-6 mannosidase and Sialidase A pre-treated pharyngeal cell surfaces. (**A**) Assay schematic for the assessment of biofilm EPS resulting from biofilm formed on each of the exoglycosidase (α1-6 mannosidase, α1-2,3 mannosidase, and Sialidase A) pre-treated pharyngeal monolayers vs. untreated. 72 h biofilms were assessed for (**B**) EPS via DMMB staining of sulphated GAGs and (**C**) EPS associated components (eDNA and protein) via fluorescent staining with Sytox Blue and FilmTracer SYPRO Ruby biofilm matrix stain, respectively. Data represents mean ± SEM, with statistical analysis performed using a one-way ANOVA with Tukey’s multiple comparisons test * (*p* ≤ 0.05) and ** (*p* ≤ 0.01); *n* = 3 biological replicates, with 3 technical replicates each.

Biofilms are comprised of bacterial cells and EPS. To further determine what may be contributing to the observed changes in biofilm biomass, EPS associated sulphated GAGs, extracellular DNA (eDNA), and protein was assessed (Figure 5A).

1,9 dimethyl methylene blue (DMMB) staining was used to detect EPS-associated sulphated GAGs [33,34]. EPS associated sulphated GAGs increased significantly for M12 GAS biofilms formed on α1-6 mannosidase (*p* ≤ 0.01) and Sialidase A (*p* ≤ 0.05) pre-treated pharyngeal cell monolayers compared to the untreated control (Figure 5B). This result supports the findings from crystal violet staining, suggesting EPS production increases in response to modification of cell surface glycans. Lastly, EPS was examined for common EPS components (eDNA and protein), via fluorescent stains Sytox Blue and SYPRO Ruby (Figure 5C). There were no significant differences in the presence of EPS associated eDNA or protein in biofilms formed.

**Figure 6:**
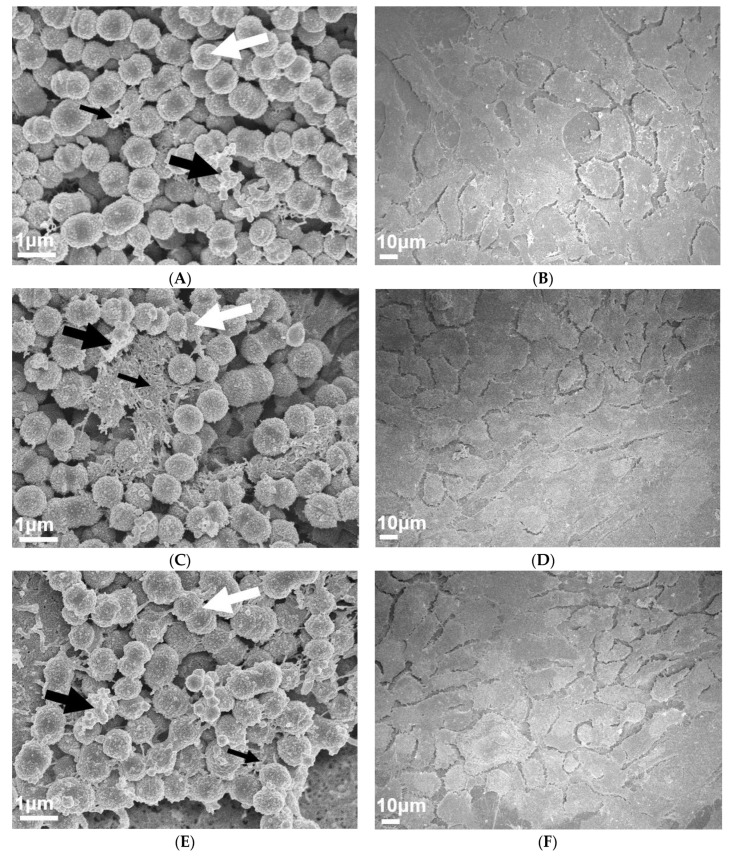

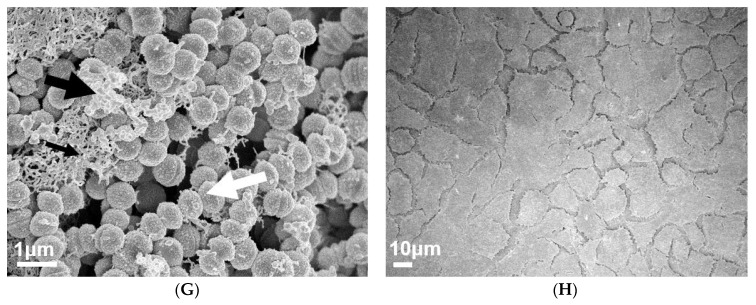
Visual inspection of 72 h M12 GAS biofilms captured via SEM revealed substantial EPS present in biofilms formed on exoglycosidase pre-treated pharyngeal cell monolayers. Images are representative of biofilms formed on (**A**) untreated, (**C**) α1-6 mannosidase, (**E**) α1-2,3 mannosidase, and (**G**) Sialidase A pre-treated pharyngeal monolayers. GAS biofilms show chained cocci (white arrows) arranged into three dimensional aggregated structures with EPS matrix material present (big and small black arrows). SEM images of (**B**) untreated, (**D**) α1-6 mannosidase, (**F**) α1-2,3 mannosidase, and (**H**) Sialidase A pre-treated Detroit 562 pharyngeal cell monolayers (without biofilm) are also included. Biofilms and Detroit 562 pharyngeal cell monolayers (without biofilm) were imaged using the JEOL JSM-7500 microscope at 15,000× and 500× magnification, respectively. SEM images were randomly selected and represent two biological replicates with two technical replicates each.

To visually investigate the effect of exoglycosidase (α1-6 mannosidase, α1-2,3 mannosidase, and Sialidase A) pre-treated pharyngeal monolayers on M12 GAS biofilm formation, SEM imaging was conducted (Figure 6). Biofilms formed on both untreated and exoglycosidase pre-treated monolayers show M12 GAS chained cocci arranged into three dimensional aggregated structures with EPS matrix material present (Figure 6A,C,E,G). Biofilms formed on each of the exoglycosidase pre-treated pharyngeal monolayers appear to produce more EPS matrix material associated with the aggregated GAS cocci when compared to GAS biofilm formed on the untreated control. EPS produced on all GAS biofilms display the distinct forms seen previously, a web-like mesh matrix (small black arrows) and a more globular matrix (big black arrows). Both extend from the cocci cell surface of these biofilm cells. Untreated and exoglycosidase pre-treated Detroit 562 pharyngeal monolayers (without biofilm) were also imaged as controls to ensure that each exoglycosidase treatment did not affect pharyngeal cell morphology/structures (Figure 6B,D,F,H).

### 2.4. Increased Biofilm Formation Promotes Penicillin Tolerance

Penicillin is the antibiotic of choice for the treatment of GAS infections [35]. The biofilm phenotype is known to decrease bacterial susceptibility to antibiotics and is suspected to play a role in the observed antibiotic treatment failure rate of 20–40% of GAS pharyngitis cases [2,3,4]. As such, the penicillin susceptibility of the M12 GAS biofilms was assessed.

Firstly, the susceptibility of planktonic M12 GAS to penicillin was confirmed by determining the minimum inhibitory concentration (MIC) and minimum bactericidal concentration (MBC) values (data not shown). A MIC and MBC of 0.025 μg/mL was established, which is consistent with the reported GAS MIC and MBC range for penicillin [36,37]. Notably, minimum biofilm eradication concentration values (MBECs) determined for M12 GAS biofilms formed on untreated and exoglycosidase pre-treated monolayers (Table 1) were all considerably higher than the MIC for planktonic M12 GAS, resulting in 2500–5000-fold greater penicillin tolerance. Specifically, biofilms formed on untreated pharyngeal monolayers had an MBEC of 62.5 μg/mL, whereas biofilms formed on exoglycosidase pre-treated monolayers had an MBEC of 125 μg/mL.

### 2.5. Targeted Removal of Glycans from Pharyngeal Monolayers Affects Biofilm Formation by GAS emm-types in both a Glycan- and Strain-Dependent Manner

The enzymatic removal of terminal mannose and sialic acid residues from the pharyngeal cell surface resulted in significant increases in M12 GAS biofilm biomass. To assess if this is the case for other GAS strains, the effect of glycan removal on biofilm formation was investigated for a diverse selection of eight GAS isolates.

**Figure 7:**
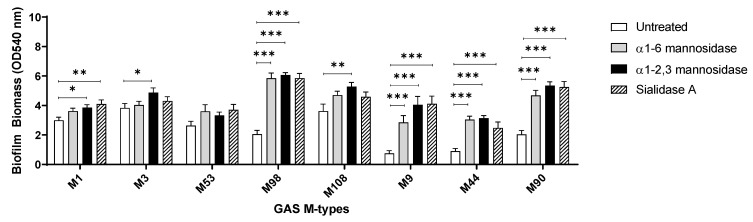
Assessment of the effect exoglycosidase treated pharyngeal cell monolayers exert on biofilms of eight other GAS M-types. Biofilm biomass was quantified via crystal violet staining. Data represents mean ± SEM, with statistical analysis performed using a one-way ANOVA with Tukey’s multiple comparisons test, * (*p* ≤ 0.05), ** (*p* ≤ 0.01), and *** (*p* ≤ 0.001); *n* = 3 biological replicates, with 3 technical replicates each.

Assessed GAS M-types (Figure 7) displayed different biofilm forming capacities on the untreated and exoglycosidase pre-treated pharyngeal monolayers. With the exception of M53, all isolates exhibited a trend of significantly increased biofilm biomass when grown on at least one of the exoglycosidase pre-treated pharyngeal monolayers. M9, M44, and M90 all displayed significant increases in biofilm biomass when grown on all three exoglycosidase pre-treated pharyngeal monolayers. Interestingly, these three M-types all belong to the same *emm* pattern (pattern E). However, no unifying trends were found when comparing GAS *emm*-types belonging to pattern A–C (M1, 3, and 12) or pattern D (M53, 98, and 108). Overall, significant increases in biofilm biomass were both strain and terminal glycan (mannose/sialic acid residue) dependent.

## 3. Discussion and Conclusions

Pharyngitis is the most prevalent form of GAS disease [1,35]. Although other pathogenic agents can cause pharyngitis, GAS is frequently isolated in affected children (20–40%) and adolescents/young adults (5–15%) [3,38]. Penicillin is the antibiotic of choice due to its narrow spectrum of activity, safety, and accessibility [35]. Most notably, there have been no reported cases of GAS resistance to penicillin to date among clinical isolates. Despite this, antibiotic treatment failure occurs in 20–40% of cases [3,39]. Numerous hypotheses have been suggested to explain this treatment failure rate including GAS host-cell internalization and viral/bacterial co-pathogenicity [40,41]. More recently, it has been proposed that GAS may exist as a biofilm, a microbial phenotype that is known to provide protection from both host immunity and antibiotics [5,42,43]. Numerous studies have since investigated biofilm formation in the context of GAS pharyngitis and treatment failure. However, many of them employ abiotic substrata, and moreover, none have considered the role of host cell surface glycans. Given the increasingly appreciated importance of glycans in the host-pathogen relationship and the abundance of glycosylated structures in the oropharynx, this work contributes to an improved understanding of the role of host glycans in the pathogenesis of GAS pharyngitis and antibiotic treatment failure.

Interactions of planktonic GAS with a variety of glycan structures have been observed in numerous studies, many of which indicate that host glycans are implicated in GAS binding and adhering to host cells [23,24,25]. In the current study, removal of *N*-linked glycans from the Detroit 562 pharyngeal cell surface significantly decreased initial adherence of planktonic M12 GAS. Despite this, subsequent M12 GAS biofilms displayed a significant increase in biofilm biomass on PNGase F treated cells. SEM imaging of biofilms formed on both untreated and PNGase F pre-treated monolayers revealed adherent M12 GAS cocci chains arranged in three dimensional aggregated structures intermeshed with EPS matrix. Moreover, biofilms formed on PNGase F pre-treated monolayers were seen to have more EPS matrix. Two varieties of EPS matrix were visible; the globular EPS matrix seen in the current study is phenotypically similar to GAS biofilms imaged via SEM previously on the surface of tonsils removed from patients with recurrent GAS tonsillopharyngitis [7]. The web-like EPS matrix projecting from the cocci cell surface of these biofilms has been captured in one previous study of M2 GAS biofilms, with the authors describing the EPS matrix material as “threadlike structures of an as-yet unknown chemical composition” [8].

Although this is an interesting finding, it is unlikely in vivo that a host cell surface would be lacking in all or most of its *N*-linked glycans. As such, we focused on the role of specific *N*-linked glycans that are abundant on the Detroit 562 pharyngeal cell surface in this study. PGC-LC-ESI-MS/MS analysis of the Detroit 562 pharyngeal cell surface *N*-glycome revealed that oligomannose structures are the most abundant class of *N*-glycan, followed by complex and hybrid glycans, with mannose being the predominant monosaccharide on the cell surface. As mannosidases act upon oligomannose *N*-glycans primarily, and hybrid *N*-glycans secondarily, broad-spectrum sialidase treatment was additionally utilised in this study to act upon complex and hybrid *N*-glycans. Removal of terminal mannose and sialic acid residues resulted in an increase of biofilm biomass similar to that induced by total *N*-glycan removal with PNGase F, independent of initial adherence and biofilm viability. It is possible that removal of these glycans enables access by GAS to otherwise impeded host receptors involved in the host-pathogen interaction, further promoting biofilm formation. The importance of terminal monosaccharides has been demonstrated in a previous study of GAS binding to human buccal epithelial (HBE) cells, whereby expression of terminal galactose and sialic acid residues had significant effects on M1, 3, and 12 associations, whilst terminal fucose and *N*-acetylgalactosamine were of comparatively lesser dependence of binding for all three GAS M-types. It was suggested that fucose and *N*-acetylgalactosamine may have a host-protective effect, sterically hindering access to the preferred galactose residues [25]. Many pathogens are known to possess their own suite of glycosidases which they utilize to liberate glycan residues for their own metabolic processes, and moreover, to better access preferred glycan structures for adherence [44,45]. For example, *S. pneumoniae* expresses multiple neuraminidases that cleave off sialic acids, unmasking other receptors for increased binding and virulence [44].

Given that initial planktonic GAS adherence and the population of live cells within the biofilms did not seem to uniquely contribute to the increases in biofilm biomass, EPS was examined. The EPS is an important component of the biofilm, and it is thought to contribute to around 80–85% of the total biofilm biomass [46]. The removal of glycans did not result in an increase of initial bacterial adherence, and it is possible changed glycan structures modified expression of genes associated with EPS production, although this was not explored in the current study. The EPS matrix has its own complex and dynamic matrixome, which defines the compositional and functional diversity of the EPS. EPS is predominantly comprised of polysaccharides, proteins, eDNA, and lipids. Despite variability in their composition across pathogens, EPS-associated polysaccharides are generally considered the most abundant [47,48,49]. Currently, GAS EPS remains poorly defined, with only one study having investigated EPS polysaccharides, determining that L-glucose and D-mannose were the most abundant sugar moieties of the EPS of one M6 strain [50].

DMMB staining is a simple approach that has been used extensively in staining the EPS GAG polysaccharides of numerous other bacterial biofilms (e.g., *Staphylococcus aureus*, *P. aeruginosa*, *Burkholderia cenocepacia*, and *Propionibacterium acnes)* [33,34]. Here, for the first time, it is demonstrated that DMMB is suitable for detecting GAS biofilm EPS-associated GAGs. Moreover, DMMB staining revealed that biofilms formed on α1-6 mannosidase and Sialidase A pre-treated pharyngeal monolayers exhibited significantly increased EPS polysaccharide production, despite eDNA or protein abundance remaining unchanged. Further assessment of other EPS components such as lipids and a diverse range of polysaccharides should be investigated via other fluorescent stains such as 1,1′-dioctadecyl-3,3,3′,3′-tetramethylindodicarbocyanine perchlorate (DiD’oil) or concanavalin A conjugated to a fluorophore such as tetramethylrhodamine [51]. SEM imaging of biofilms formed on both untreated and each of the exoglycosidase pre-treated monolayers revealed biofilms similar to those seen on untreated and PNGase F pre-treated monolayers. Biofilms show adherent M12 GAS cocci chains arranged in three dimensional aggregated structures intermeshed with EPS matrix. Similar to results following PNGase F treatment, biofilms formed on exoglycosidase pre-treated pharyngeal cell monolayers showed an increase in EPS matrix. Both varieties of EPS (globular and web-like) were present in biofilms formed.

A critical characteristic of the biofilm phenotype is increased tolerance to antimicrobials, with reports of antimicrobial tolerance anywhere between 10 and >1000 times greater compared to planktonic form [52,53,54]. Biofilms may be a contributing factor for the antibiotic treatment failure rate of 20–40% reported in cases of GAS pharyngitis. Here we determined if the observed increase in biofilm formation was functionally relevant in the context of penicillin tolerance when compared to planktonic GAS. Penicillin tolerance doubled following removal of mannose and sialic acid residues, respectively, from the pharyngeal monolayer substratum. We have shown that removal of these residues leads to increases in EPS which may have impeded penicillin penetration through the biofilm. Several other features unique to the biofilm phenotype may have further contributed to the increase in penicillin tolerance including differential growth rates of bacterial cells, nutrient gradients, and antibiotic degradation once in the biofilm [55,56,57,58]. Further study should aim to characterise the specific mechanisms for the enhanced penicillin tolerance described here.

The indiscriminate removal of all *N*-glycans and the targeted removal of terminal mannose and sialic acid, which predominate the surface of Detroit 562 pharyngeal cells, increases biofilm biomass, specifically via increased EPS production. Similar results were observed in eight other clinically relevant GAS *emm*-types, albeit in a glycan- and strain-dependent manner, suggesting that the influence of the host glycome on the biofilm phenotype is not limited to a single strain or *emm*-type. The presence of certain host glycan structures may be a host-protective mechanism, reducing the formation of GAS biofilms in vivo. GAS has been shown to modify host glycoproteins via recently discovered glycosidases endoglycosidase S and α-mannosidase [59,60]. The ability of GAS to modify the host-glycome at different sites and stages of infection has not been characterised. Our findings suggest that modification of the host glycome during the course of infection may increase the ability of GAS to form biofilms.

GAS is commonly attributed to pharyngeal infection with over 600 million cases per year, presenting a considerable global burden [1]. Recurrent GAS infection, persistent carriage, and antibiotic treatment failure remain challenging and unresolved, despite numerous efforts in characterising the consortia of molecular mechanisms underpinning GAS virulence and pathogenesis. Moreover, most of these studies have been conducted only in the context of planktonic GAS. Here GAS biofilm formation has been investigated, with a particular focus on the effect of altering pharyngeal cell surface glycans. Host cell surface glycans may offer a protective advantage against GAS biofilm formation. We have shown that modulation of the pharyngeal glycome has a direct impact on GAS biofilm formation, with increases in EPS likely to play an important role. Moreover, the increased GAS biofilms displayed significantly greater penicillin tolerance dependent on the host cell glycome. This study describes the effect of host glycosylation on GAS biofilm formation and GAS biofilm formation as an important proponent in penicillin tolerance.

## 4. Materials and Methods

### 4.1. GAS and Culture Conditions

GAS strains used in this study (see Supplementary Material, Table S2) are clinical GAS isolates, with each strain representative of a discrete GAS *emm*-type [25,61,62,63,64]. GAS was grown on horse blood agar (HBA) plates (Edwards, Murarrie, Australia) or Todd Hewitt agar supplemented with 1% (*w*/*v*) yeast (THYA). Static cultures of GAS were grown overnight in Todd Hewitt broth (BD Sparks, MD, USA) supplemented with 1% (*w*/*v*) yeast (THY). GAS was cultured and maintained at 34 °C [65].

### 4.2. Human Pharyngeal Cell Culture Conditions and Monolayer Formation

Detroit 562, a human pharyngeal epithelial cell line (CellBank Australia, Westmead, Australia), was cultured in Dulbecco’s Modified Eagle Medium (DMEM) F12 (Gibco, Grand Island, NY, USA), supplemented with 2 mM L-glutamine (Gibco, Life Technologies, Grand Island, NY, USA) and 10% (*v*/*v*) heat inactivated foetal bovine serum (FBS) (Bovogen Biologicals, Keilor East, Australia) in cell culture flasks at 37 °C, 5% CO_2_ to 20% O_2_ atmosphere.

Fixed Detroit 562 pharyngeal cell monolayers form the substratum for bacterial growth for subsequent biofilm experiments. In brief, wells of 96-well flat bottom cell culture microtiter plates (Greiner Bio-One, Frickenhausen, Germany) were coated with 300 µg/mL Collagen I from rat tail (Gibco, Life Technologies, Grand Island, NY, USA) and incubated for 1 h, 37 °C, 5% CO_2_ to 20% O_2_ atmosphere. After 1 h, wells were seeded with 150 µL Detroit 562 cell suspension (2 × 10^5^ cells/mL) and cultured for 48 h (or until a monolayer of ~95% confluency was achieved). Monolayers were washed once with PBS and fixed with sterile 3.7% paraformaldehyde for 20 min. Once fixed, wells were washed twice with PBS, and monolayers were kept wet via submersion in PBS until required for use.

### 4.3. Characterisation of Detroit 562 Pharyngeal Cell Surface N-linked Glycans

Cell surface membrane proteins were purified from Detroit 562 culture lysates as previously described [30]. *N*-glycans released by PNGase F (Promega, Madison, WI, USA) were purified and processed, prior to analysis by porous graphitised carbon liquid chromatography coupled with electrospray ionisation tandem mass spectrometry as described elsewhere [66]. For methods in full, see Supplementary Material 3.

### 4.4. Detroit 562 Pharyngeal Cell Monolayer Pre-Treatment

#### 4.4.1. PNGase F Treated Monolayers

Wells of the 96-well microtiter plates containing pre-formed fixed Detroit 562 pharyngeal cell monolayers were blocked with 1% PVP40 solution and incubated for 5 min. Once blocked, the PVP40 was removed, and the wells washed thrice with water. For the removal of *N*-linked glycans, 5 µL of PNGase F (50 U) (Promega, Madison, WI, USA) and 10 µL PBS was added to each well and incubated overnight at 37 °C. After incubating with PNGase F, released *N*-linked glycans were removed and the wells washed once with PBS. Untreated PBS wells representing the whole glycome of the Detroit 562 pharyngeal cell monolayers were also included as a control. *N*-linked glycan removal was confirmed via Concanavalin A Alexa fluor 647 lectin binding assay (Supplementary Material, Figure S1). In brief, untreated and PNGase F pre-treated pharyngeal cell monolayers were incubated with 5 µg/mL Concanavalin A Alexa fluor 647 (Life technologies, Carlsbad, CA, USA) for 15 min (RT, dark). Unbound lectin removed, and monolayers washed twice with 100 µL PBS. Untreated and PNGase F pre-treated monolayers incubated without lectin (PBS) served as background/auto-fluorescence controls and were subtracted from sample reads. Samples were read spectrofluorometrically at excitation 625–30 nm/emission 680–30 nm.

#### 4.4.2. Exoglycosidase: α1-6 Mannosidase, α1-2, 3 Mannosidase, and Sialidase A Treated Monolayers

Pre-formed fixed Detroit 562 pharyngeal cell monolayers of the 96-well microtiter plate were treated with 30 µL/well reaction volumes of each of the exoglycosidases. For mannosidases, the reaction volume comprises 3 µL 1 × GlycoBuffer 1 (NEB, Notting Hill, Australia), 0.3 µL 100 µg/mL purified BSA (NEB, Notting Hill, Australia), 0.2 µL α1-6 mannosidase (8 U) or α1-2,3 mannosidase (8 U) (NEB, Notting Hill, Australia), and 26.5 µL PBS. For Sialidase A, 6 µL 5 × Reaction Buffer B (250 mM sodium phosphate pH 6.0) (Prozyme, Hayward, CA, USA), 0.2 µL Sialidase A (1 × 10^−3^ U) (Prozyme, Hayward, CA, USA), and 23.8 µL PBS. Untreated PBS wells representing the intact surface glycome of the fixed Detroit 562 pharyngeal cell monolayers were also included as a control. The plate was incubated for 2 h, 37 °C. Once incubated, the wells were washed once with PBS. Glycan removal was confirmed via lectin binding assay as per [25], with exoglycosidase pre-treated pharyngeal cell monolayers incubated with either biotinylated *Hippeastrum hybrid* lectin (binding mannose residues) (Vector Laboratories, Burlingame, CA, USA) or biotinylated *Sambucus nigra* lectin (binding sialic acid residues) (Vector Laboratories, Burlingame, CA, USA) (Supplementary Material 5, Figure S2).

### 4.5. Initial Adherence of Planktonic GAS

Pre-formed fixed Detroit 562 pharyngeal cell monolayers were inoculated with 150 µL of stationary phase GAS culture diluted 1:20 in THY (*v*/*v*) supplemented with sterile 0.5% glucose (THY-G) and incubated for 2 h to promote initial attachment (34 °C, slow shaking at 50 rpm). At 2 h, non-adherent GAS was removed, and the wells washed thrice with PBS. To detach Detroit 562 cells from the bottom of the microtiter plate, 0.05% trypsin-EDTA (1×) (Gibco, Grand Island, NY, USA) was added to each well and incubated (15 min, 37 °C). To lyse the now-detached Detroit 562 cells containing internalised bacteria, 0.025% Triton X-100 was added and pipetted vigorously. To enumerate the adherent GAS population, 10-fold serial dilutions of the cell suspensions were performed in PBS and aliquots spot plated onto THYA (incubated overnight, 34 °C) for subsequent colony counting and CFU/mL determination.

### 4.6. GAS Biofilm

96-well microtiter plates containing pre-formed fixed Detroit 562 pharyngeal cell monolayers untreated and pre-treated with either PNGase F or the exoglycosidases were inoculated with 150 µL of overnight GAS culture diluted 1:20 in THY-G. The inoculum was incubated for 2 h (34 °C, 50 rpm). At 2 h, non-adherent GAS was removed, and wells replenished with sterile THY-G. Subsequent 72 h GAS biofilms were produced (34 °C, 50 rpm), with sterile THY-G media refreshment performed every 24 h.

#### 4.6.1. GAS Biofilm Biomass Crystal Violet Staining

Biofilm biomass was assessed via crystal violet (CV) staining. Biofilms were air dried for 30 min (or until completely dried), and fixed with 99% methanol for 15 min. Once fixed, the biofilms were thoroughly air-dried and stained with 0.2% CV (*w*/*v*) (Sigma-Aldrich, St Louis, MO, USA) supplemented with 1.9% ethanol (*v*/*v*) for 10 min (RT, static). Once stained, excess CV was removed and each well gently washed twice with PBS. CV stain that had incorporated into the biofilm was re-solubilised in 1% sodium dodecyl sulphate (SDS) (*w*/*v*) (Sigma-Aldrich, St Louis, MO, USA), and incubated (10 min, RT). Monolayers with THY-G (no GAS biofilm) served as media sterility controls and background staining controls, with absorbance values subtracted from those of biofilm samples. Biofilm biomass quantification was performed by diluting the released dye 1:5 in the 1% SDS solution, and subsequently measured at OD_540nm_ using a SpectraMax Plus 384 microplate reader.

#### 4.6.2. Enumeration of Live Cells within GAS Biofilm

GAS biofilms were assessed for the live cell populations via enumeration of serially diluted biofilms. Briefly, biofilms were washed once in PBS and thoroughly re-suspended in fresh PBS via vigorous scraping of biofilms from the well surface, followed by a 5 min sonication. The population of live cells within these biofilms were enumerated via 10-fold serially diluting in PBS, and spot plating onto THYA (incubated overnight, 34 °C) for subsequent colony counting and CFU/mL determination.

#### 4.6.3. GAS Biofilm EPS

To assess biofilm EPS, the (i) EPS-GAGs and (ii) common EPS components (eDNA and protein) were examined. Briefly, EPS associated GAGs were quantified by 1,9-dimethyl methylene blue (DMMB) dye based EPS assay adapted from methods described elsewhere [33]. EPS components (eDNA and protein) were fluorescently stained with Sytox Blue, a cell membrane-impermeant nucleic acid stain (Molecular Probes, Invitrogen, Eugene, OR, USA) and FilmTracer SYPRO Ruby biofilm matrix stain that labels most classes of proteins (Molecular Probes, Invitrogen, Eugene, OR, USA). In brief, biofilms were fixed with 99% methanol for 15 min, and subsequently air dried. Biofilms were individually stained for 30 min in the dark with 5 µM Sytox Blue and 0.5× concentration FilmTracer SYPRO Ruby biofilm matrix stain, with PBS as a control. Fixed Detroit 562 pharyngeal monolayers without biofilm were also stained and served as controls for background staining, with resultant absorbance values subtracted from biofilm EPS measurements. Fluorescence was measured using a CLARIOStar (with 6 × 6 matrix well scanning of the non-homogenous biofilms). Sytox Blue was read with an excitation of 440–15 nm and emission of 484–20 nm, and FilmTracer SYPRO Ruby biofilm matrix read with an excitation of 450–15 nm and emission of 610–20 nm.

### 4.7. M12 GAS Penicillin Susceptibility

As per accepted guidelines and standard protocols the MIC and MBC values were determined for penicillin activity against planktonic M12 GAS [67,68]. Briefly, M12 planktonic GAS suspension (1 × 10^6^ CFU/mL) was challenged with serially diluted penicillin and incubated (24 h, 34 °C). The MIC was defined as the lowest concentration of penicillin required to completely inhibit bacterial growth (indicated by clear wells), and further confirmed by measuring OD_600nm_ using a SpectraMax Plus 384 microplate reader. MBC was defined as the lowest concentration of penicillin required to induce complete killing of bacteria as determined upon spot plating on THYA. For biofilm susceptibility, MBEC was determined by challenging the pre-formed biofilms with 2-fold dilutions of penicillin in THY, 2% G (*v*/*v*) (24 h, 34 °C). Biofilms were washed once in PBS and thoroughly re-suspended in fresh PBS. Viable cells were enumerated via 10-fold serially diluting in PBS, and spot plating onto THYA (incubated overnight, 34 °C) for subsequent colony counting and CFU/mL determination. The MBEC was determined as the lowest concentration of penicillin required to induce complete eradication of GAS biofilm [69].

### 4.8. Scanning Electron Microscopy

M12 GAS biofilms were grown on untreated and PNGase F or exoglycosidase pre-treated fixed Detroit 562 pharyngeal monolayers on 13 mm plastic Nunc Thermanox coverslips (Proscitech, Rochester, NY, USA) in a 12-well polystyrene plate. Biofilms were air dried, and prepared for SEM using methods adapted from [70] with the following modifications. In brief, biofilms were pre-fixed in 2.5% glutaraldehyde (Sigma-Aldrich, St Louis, MO, USA), 50 mM L-lysine monohydrochloride, and 0.001% ruthenium red (Sigma-Aldrich, St Louis, MO, USA) solution prepared in 0.1 M HEPES buffer (pH 7.3) (30 min, 4 °C). Following pre-fixation, biofilms were fixed in fixative solution (2.5% glutaraldehyde and 0.001% ruthenium red prepared in 0.1 M HEPES buffer, pH 7.3) for 1.5 h (4 °C) and washed twice in 0.1 M HEPES buffer. Post-fixation (2 h), 2% osmium tetroxide vapour was used, followed by three washes with distilled water (each 15 min). A graded ethanol series (30%, 50%, 70%, 90%, and 3 × 100%) was then used to remove all water from the biofilms before they were critical point dried (Leica CPD 030, Austria). Dried biofilms were then sputter coated with 20 nm platinum (Edwards Vacuum coater, USA) and visualized using a JEOL JSM-7500 microscope (JEOL, Japan) at 15,000× magnification. Untreated and PNGase F or exoglycosidase pre-treated fixed Detroit 562 pharyngeal monolayer controls (without biofilms) were also imaged at 500× magnification. Images were taken at random positions within the samples by an UOW Electron Microscopy Centre technician blinded from the study in an effort to reduce bias.

### 4.9. Statistical Analysis

All statistical analysis was performed using GraphPad Prism (version 8.4.0, GraphPad Software, USA). A student’s t-test or a one-way ANOVA was performed with a Tukey’s multiple comparisons post hoc test where relevant. A *p*-value of ≤ 0.05 was considered significant.

## Figures and Tables

**Table 1:** M12 GAS biofilms exhibit enhanced penicillin tolerance when formed upon exoglycosidase-treated pharyngeal cell monolayers. MBECs (μg/mL) determined for M12 GAS biofilms were compared to the planktonic M12 GAS minimum inhibitory concentration (MIC) (μg/mL). Data represent *n* = 3 biological replicates, with 3 technical replicates each.

Biofilm Monolayer	M12 MBEC (μg/mL)	Fold Greater Tolerance Compared to MIC
Untreated	62.5	2500
α1-6 mannosidase	125	5000
α1-2,3 mannosidase	125	5000
Sialidase A	125	5000

## Supplementary Materials

The following are available online at https://www.mdpi.com/2079-6382/9/11/775/s1. Table S1: Proposed *N*-glycan structures identified by PGC-LC-ESI-MS/MS following PNGase F treatment of membrane proteins extracted from Detroit-562 pharyngeal cells. Table S2: GAS strains utilised in this study, their M-types and clinical source.Method Text 1 S3: Characterisation of the Detroit 562 pharyngeal cell surface *N*-linked glycans. Figure S1: Lectin binding assay confirming removal of *N*-linked glycans via PNGase F treatment of Detroit 562 pharyngeal cell monolayers. Figure S2: Lectin binding assay confirming removal of terminal mannose and sialic acid residues via exoglycosidase treatment of Detroit 562 pharyngeal cell monolayers.
