# Supplementary material for: Assessing the Role of Pharyngeal Cell Surface Glycans in Group A Streptococcus Biofilm Formation
Source: Antibiotics (Basel). 2020 Nov 4;9(11):775. doi: 10.3390/antibiotics9110775 (PMC7694240; doi:10.3390/antibiotics9110775)
Supplement: Supplementary file 1 [file antibiotics-09-00775-s001.pdf]

Supplementary Materials

Assessing the role of pharyngeal cell surface glycans in Group A *Streptococcus* biofilm formation

Heema K. N. Vyas[1,2], Anuk D. Indraratna[1,2], Arun Everest-Dass[3], Nicolle H. Packer[3,4], David M. P. De Oliveira[5], Marie Ranson[1,2], Jason D. McArthur[2], Martina L. Sanderson-Smith[1,2*]

[1] Illawarra Health and Medical Research Institute, Wollongong, New South Wales, Australia.
[2] School of Chemistry and Molecular Bioscience, Molecular Horizons, University of Wollongong, Wollongong, New South Wales, Australia.
[3] Institute for Glycomics, Griffith University, Gold Coast Campus, Southport, Queensland, Australia.
[4] Department of Molecular Sciences, Macquarie University, Sydney, New South Wales, Australia.
[5] School of Chemistry and Molecular Biosciences, University of Queensland, Brisbane, Queensland, Australia.
* Correspondence: martina@uow.edu.au; Tel.: +61 2 42981935 (NSW, Australia)

Supplementary Material 1:

Table 1. Proposed *N*-glycan structures identified by PGC-LC-ESI-MS/MS following PNGase F treatment of membrane proteins extracted from Detroit 562 pharyngeal cells. Structures are assigned based on precursor m/z, retention time and MS^2 fragmentation spectra [28-31]. Relative abundances were calculated based on integration of peak area of extracted ion chromatograms and reported as mean ± SEM across three biological replicates. Abundances of structural isomers sharing the same m/z, monosaccharide composition and terminal monosaccharide presentation have been combined for clarity. Glycans are represented using conventional graphical nomenclature as per [32]. Red triangle = fucose (dHex), yellow circle = galactose (GalNAc), blue square = *N*-acetylglucosamine (GlcNAc), green circle = mannose (Man), purple diamond = sialic acid (NeuAc).

#	m/z [M-H]$^{-2}$	m/z [M-H]$^{-1}$	Composition	Terminal mono-saccharide	Proposed structure	Structural isomers	Relative abundance (%)
1)	617.2	1235.4	(Man)$_5$ (GlcNAc)$_2$	Mannose	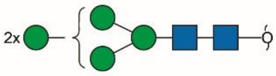	4	1.90 ± 0.04
2)	698.2		(Man)$_6$ (GlcNAc)$_2$	Mannose	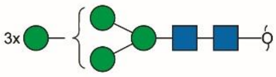	3	17.43 ± 0.36
3)	771.3		(Man)$_6$ (GlcNAc)$_2$ (dHex)$_1$	Mannose	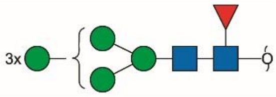	2	1.07 ± 0.12
4)	779.3		(Man)$_7$ (GlcNAc)$_2$	Mannose	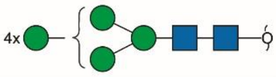	3	25.36 ± 1.24
5)	820.3		(Man)$_3$ (GlcNAc)$_4$ (Gal)$_2$	Galactose	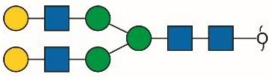	2	0.66 ± 0.06
6)	860.3		(Man)$_8$ (GlcNAc)$_2$	Mannose	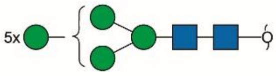	2	26.05 ± 0.54
7)	893.3		(Man)$_3$ (GlcNAc)$_4$ (Gal)$_2$ (dHex)$_1$	Galactose	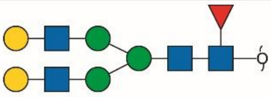	2	1.24 ± 0.07
8)		895.3	(Man)$_2$ (GlcNAc)$_2$ (dHex)$_1$	Mannose	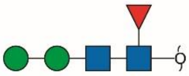	2	1.53 ± 0.04
9)	937.7		(Man)$_4$ (NeuAc)$_1$ (GlcNAc)$_3$ (Gal)$_1$ (dHex)$_1$	Mannose Sialic acid	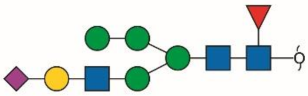	3	1.50 ± 0.09
10)	941.3		(Man)$_9$ (GlcNAc)$_2$	Mannose	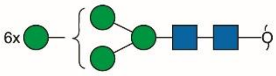	3	7.77 ± 0.91
11)	945.3		(Man)$_5$ (NeuAc)$_1$ (GlcNAc)$_3$ (Gal)$_1$	Mannose Sialic acid	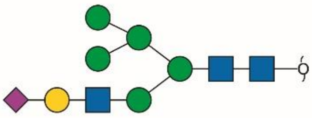	2	2.50 ± 0.51

12)	957.8		$(Man)_3$ $(NeuAc)_1$ $(GlcNAc)_4$ $(Gal)_1$ $(dHex)_1$	GlcNAc Sialic acid	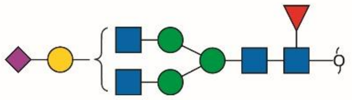	3	0.55 ± 0.14
13)	965.8		$(Man)_3$ $(NeuAc)_1$ $(GlcNAc)_4$ $(Gal)_2$	Galactose Sialic acid	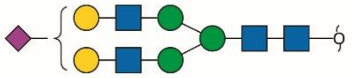	3	2.31 ± 0.41
14)	1018.4		$(Man)_5$ $(NeuAc)_1$ $(GlcNAc)_3$ $(Gal)_1$ $(dHex)_1$	Mannose Sialic acid	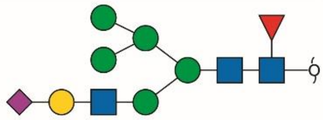	2	1.24 ± 0.24
15)	1038.9		$(Man)_3$ $(NeuAc)_1$ $(GlcNAc)_4$ $(Gal)_2$ $(dHex)_1$	Galactose Sialic acid	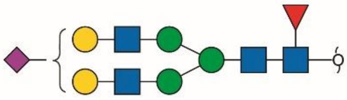	4	5.63 ± 0.86
16)	536.15	1073.3	$(Man)_4$ $(GlcNAc)_2$	Mannose	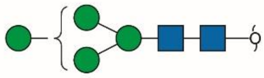	3	0.99 ± 0.33
17)	1111.4		$(Man)_3$ $(NeuAc)_2$ $(GlcNAc)_4$ $(Gal)_2$	Sialic acid	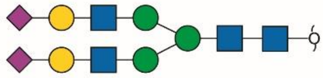	2	0.63 ± 0.27
18)	1111.9		$(Man)_3$ $(NeuAc)_1$ $(GlcNAc)_4$ $(Gal)_2$ $(dHex)_2$	Galactose Sialic acid Fucose	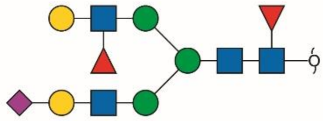	2	0.56 ± 0.06
19)	1184.3		$(Man)_3$ $(NeuAc)_2$ $(GlcNAc)_4$ $(Gal)_2$ $(dHex)_1$	Sialic acid	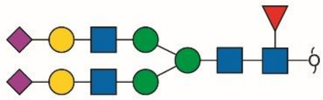	3	1.22 ± 0.06

Supplementary Material 2:

Table 2. GAS strains utilised in this study, their M-types and clinical source [23,24,61-64].

M-type	Clinical source
**M12**	**Superficial infection: persistent Pharyngeal pus/sinusitis**
M1T1	Invasive infection: Necrotizing fasciitis and toxic shock
M3	Invasive infection
M53	Invasive infection: Blood
M98	Invasive infection: Blood (bacteraemia)
M108	Superficial infection: Wound
M9	Invasive infection: Pustules on foot (bacteraemia)
M44 (/61)	Superficial infection: Skin sore
M90	Invasive infection: Necrotising fasciitis, hip pus

Supplementary Material 3:

Characterisation of the Detroit 562 pharyngeal cell surface *N*-linked glycans

Characterisation of the Detroit 562 pharyngeal cell surface *N*-linked glycans purified from cell surface glycoproteins was conducted as per the methods developed by Everest-Dass, Jin, Thaysen-Andersen, Nevalainen, Kolarich and Packer [30] with slight modification. In brief, 3 different passages derived from the same vial of the Detroit 562 cells (representative of 3 biological replicates) were pelleted and washed once in 14 mL PBS by centrifugation (20 min, 1000g, 4°C) in a Heraeus Multifuge X3R centrifuge (Thermofisher Scientific, USA). Cells were counted using a haemocytometer and cell concentration adjusted to $2x10^8$ cells/mL and pelleted by centrifugation (5 min, 1000g, 4°C). The pellet was re-suspended in 2 mL of lysis buffer and thoroughly homogenised using a Branson 250 digital sonicator (Branson, USA) (30% amplitude, 6 watts, 5-s pulses for 2 min) followed by syringe lysis with a 23-gauge needle. Supernatant containing the cellular proteins was collected from the centrifuged homogenate and further diluted with lysis buffer and sedimented by ultracentrifugation (1 h, 100 000g, 4°C) with a Sorvall MTX 150 Micro-Ultracentrifuge (Thermofisher Scientific, USA) [71]. Resultant membrane protein pellet was re-suspended in 1 mL lysis buffer containing 1% (v/v) Triton X-114, and the pellet completely homogenized. Samples were heated at 37°C for 20 min, and phase partitioned by centrifugation (5 min, 300 g, RT). The Triton X-114 layer containing the membrane proteins were precipitated with 9 volumes of ice-cold acetone and incubated overnight at -20°C. The precipitated membrane proteins were re-solubilised in 100 µL of 8 M urea, and protein quantified by DC assay. 20 µg of protein lysate from each of the 3 passages were independently dot blotted in triplicate onto EtOH-wetted PVDF membrane (20 µg of BSA and bovine fetuin were included as glycan negative and positive controls respectively) and dried overnight (RT). Dried dot blots were then washed in MeOH (15 min, shaking, RT) and further washed in water (15 min, shaking, RT). To qualitatively confirm and visualise immobilised protein, the dot blotted membrane was stained with Coomassie blue for 3 min, and then de-stained, and the membrane placed in water. Protein-laden spots of 6mm diameter were excised from the PVDF membrane and submerged into wells containing 100 µL 1% PVP40 where they were incubated for 5 min, and then washed thrice with 200 µL water. For the removal of *N*-linked glycans, 2 µL of PNGase F (1000 U) (NEB) and 8 µL water was added to each well and incubated overnight at 37°C. After incubating overnight, the 96-well plate was sonicated for 5 min in an Ultrasonic Cleaner sonicating water bath (Unisonics, Australia). The wells were then rinsed with 20 µL water, and the solutions collected. The samples were then acidified with 10 µL 100 mM ammonium acetate (pH 5) and incubated for 1 h, RT. Released *N*-glycans were reduced in alkaline conditions by the addition of 20 µL of 1.25 M $NaBH_4$ in 100 mM KOH, incubated for 3 h at 50°C. After cooling to RT, the reaction was neutralised with 2 µL of glacial acetic acid.

Sodium salts of *N*-glycan solutions were removed by passage through and subsequent washing of cation exchange microcolumns constituted by packing 25 µL of Dowex® 50W X8 (Sigma-Aldrich, USA) in a ZipTip® C18 tip (Merck Millipore), activated with 50 µL of 1 M HCl prior to addition of sample. Solutions were passed through columns using a benchtop micro-centrifuge at full speed for 15-30 s. Sodium-desalted samples were vacuum dried in a Savant SPD131DDA SpeedVacTM Concentrator (ThermoFisher). Samples were then washed and dried three times with 100 µL of methanol to remove residual borate.

Desalted samples were further purified by porous graphitised carbon (PGC) chromatography. Columns were constituted with 5 µL of PGC material from HyperSep Hypercarb cartridges (ThermoFisher) in methanol, deposited in a ZipTip® C18 tip. Prior to addition of sample, columns were washed with elution buffer (80% v/v acetonitrile (ACN), 0.1% v/v trifluoroacetic acid (TFA)) and subsequently equilibrated in loading buffer (0.1% v/v TFA). Dried sample was dissolved in 50 µL loading buffer and passed through columns, with flow through re-loaded into the columns. Enriched glycans were eluted in 50 µL of elution buffer and vacuum dried. Dried, purified enriched glycans were stored at –20 °C.

Dried glycans were reconstituted in 20 μL of water immediately prior to LC-MS/MS analysis. Sample handling and injections were performed using an Ultimate 3000 UHPLC (Thermo Scientific). Samples were injected in running buffer (10 mM NH_4HCO_3) through a PGC pre-column (3 μm Hypercarb, 320 μm ID x 100 mm) at a flow rate of 6 μL/min and subsequently a PGC analytical column (3 μm Hypercarb, 75 μm ID x 100 mm) at a flow rate of 1 μL/min. Chromatographic separation was achieved using a 120 min gradient for *N*-glycans (0 – 70% v/v ACN). The HPLC system was directly connected to an electrospray ionisation source (AmaZon Speed Ion-Trap, Bruker Corporation, UK) with a capillary voltage of 3 kV. MS spectra were obtained in negative mode between m/z 400 and 1800 with an accumulation time of 200 ms.

Data analyses were carried out in Compass Data Analysis 4.2 (Bruker Corporation) for structural assignment and in Skyline 20.1 [72] for quantitation. Quantitation was performed for each isomer identified by MS^2 spectra and retention time by calculating the area under the curve (AUC). This was expressed as a percentage of total AUC yielding relative abundance for each replicate.

Supplementary Material 4:

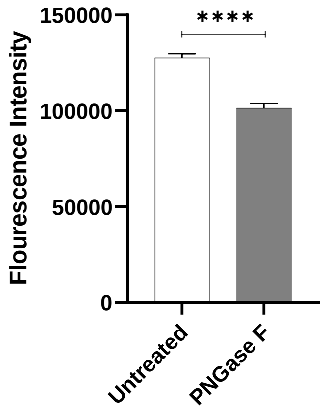

Fig 1. Lectin binding assay confirming removal of *N*-linked glycans via PNGase F treatment of Detroit 562 pharyngeal cell monolayers. *N*-linked glycan removal was confirmed via Concanavalin A Alexa Fluor 647 lectin binding to untreated and PNGase F pre-treated pharyngeal cell monolayers. Bound Concanavalin A Alexa Fluor 647 was detected spectrofluorometrically at excitation 625-30 nm/emission 680-30 nm. Data represents mean ± SEM, with statistical analysis performed, **** ($P \leq 0.0001$); n = 3 biological replicates, with 2 technical replicates each.

Supplementary Material 5:

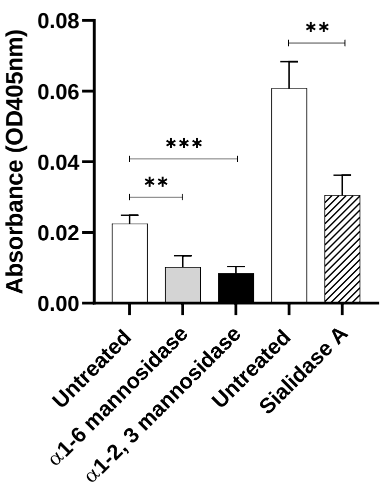

Fig 2. Lectin binding assay confirming removal of terminal mannose and sialic acid residues via exoglycosidase treatment of Detroit 562 pharyngeal cell monolayers. Glycan removal was confirmed via lectin binding assay with α1-6 mannosidase and α1-2,3 mannosidase pre-treated pharyngeal cell monolayers incubated with biotinylated *Hippeastrum Hybrid* lectin (binding mannose residues), and Sialidase A pre-treated pharyngeal cell monolayers incubated with biotinylated *Sambucus negra* lectin (binding sialic acid residues). Untreated pharyngeal cell monolayer controls were incubated with respective lectins. Data represents mean ± SEM, with statistical analysis performed, ** ($P \leq 0.01$) and *** ($P \leq 0.001$); n = 5 biological replicates, with 3 technical replicates each.
